# Oxidative Stress and Nuclear Reprogramming: A Pilot Study of the Effects of Reactive Oxygen Species on Architectural and Epigenetic Landscapes

**DOI:** 10.3390/ijms24010153

**Published:** 2022-12-21

**Authors:** Claudio Casali, Stella Siciliani, Luca Galgano, Marco Biggiogera

**Affiliations:** 1 Laboratory of Cell Biology and Neurobiology, Department of Biology and Biotechnology “L. Spallanzani”, University of Pavia, 27100 Pavia, Italy; 2 Laboratory of Biochemistry, Department of Biology and Biotechnology “L. Spallanzani”, University of Pavia, 27100 Pavia, Italy

**Keywords:** oxidative stress, epigenetics, gene expression regulation, chromatin remodeling, histone post-translational modification, EZH2, potassium bromate, antioxidants

## Abstract

Cell genome integrity is continuously threatened by various sources, both endogenous and exogenous. Oxidative stress causes a multitude of damages, severely affecting cell viability, fidelity of genetic information inheritance, and determining profound alterations in gene expression. Epigenetics represents a major form of gene expression modulation, influencing DNA accessibility to transcription factors and the overall nuclear architecture. When assessing the stress-induced epigenome reprogramming, widely diffused biochemical and molecular approaches commonly fail to incorporate analyses such as architectural chromatin alterations and target molecules precise spatial localization. Unveiling the significance of the nuclear response to the oxidative stress, as well as the functional effects over the chromatin organization, may reveal targets and strategies for approaches aiming at limiting the impact on cellular stability. For these reasons, we utilized potassium bromate treatment, a stressor able to induce DNA damages without altering the cellular microenvironment, hence purely modeling nuclear oxidative stress. By means of high-resolution techniques, we described profound alterations in DNA and histone epigenetic modifications and in chromatin organization in response to the reactive oxygen species.

## 1. Introduction

Free radicals effect on organisms is a central topic in biology and it is intensively studied because of its importance in several fields, such as cellular signaling, ageing, and human pathologies [1]. In case of imbalance in the redox homeostasis, an oxidative stress condition is established with deleterious effects on biomolecules and detrimental consequences on cells and organisms [2]. High ROS levels, capable of overwhelming cellular antioxidant defenses, can be determined by exogenous sources, such as radiations, anticancer drugs and transition or heavy metals, or by endogenous causes [3].

ROS are important players in the initiation and progression of multiple forms of cancer and inflammatory diseases [4]. Furthermore, high levels of ROS have been found in metabolic diseases such as obesity and type 2 diabetes (T2D), whose complexity is reflected by the many organs and tissues contemporaneously affected; in endocrine diseases, such as Cushing’s syndrome; in autoimmune thyroid diseases: for instance, Hashimoto’s thyroiditis and Graves’ disease have been associated to oxidative stress, but also alteration in the pattern of DNA methylation. Overall, ROS may contribute to the pathogenesis through both genetic and epigenetic mechanisms, for example by altered gene expression modulation due to hypermethylation or hypomethylation. For these reasons, oxidative stress may represent a relevant therapeutic target, as antioxidants may effectively be beneficial in reducing the damage extent to biomolecules and, consequently, the pathogenesis of several diseases [5,6,7,8,9]. Despite the advances in understanding the effects of common epigenetic mechanisms on nuclear DNA, the link with oxidative stress remains poorly understood. Oxidative stress can be modelled using different experimental approaches, such as ionizing radiations, genetic manipulations of antioxidant defenses, or direct exposure of cells or animals to chemicals [10]. Whereas all these methods are considered useful tools to investigate DNA damage, a large scale of systemic damages and molecular alterations are unintentionally triggered, preventing the possibility to address the question of how cells respond to an oxidative insult under more physiological conditions. For instance, microirradiation induces high local damage concentration, while the largely used ROS-inducing compound menadione can determine high toxicity, broadly impacting cell viability and stress response. In this context, the oxidizing agent potassium bromate (KBrO_3_) represents a powerful approach to model oxidative stress without altering the cellular microenvironment, considering its lower cellular toxicity and lack of compensatory effects on cell processes [11,12,13]. Despite being classified as a possible human carcinogen (group 2B) by the International Agency for Research on Cancer (IARC), in certain countries potassium bromate is still used as food additive in bread, beer and cheese industry, as well as in cosmetic and pharmaceutical industries. KBrO_3_ can induce multiple organ toxicity, especially targeting endocrine system cells such as thyroid and renal tissue [14]. From a molecular point of view, KBrO_3_ is known to induce 8-oxoG in genomic DNA, while other DNA lesions such as endonuclease III sensitive sites, SSBs and abasic sites are formed only in a smaller proportion [15]. However, common molecular and biochemical approaches often lack the capability to consider the various degree of nuclear DNA condensation determined by the chromatin structure and by the influence of epigenetic modifications [16,17,18,19].

The epigenetic machinery is extremely complicated and heterogeneous. One of the main gene expression regulation mechanisms is represented by DNA methylation. According to the most diffused interpretation, DNA methylation in the form of 5-methylcytosine (5mC) is a hallmark of gene repression, by altering the binding affinity of transcription factors and/or affecting chromatin structure, reducing nucleosome flexibility, and determining a more compact environment, which in turn negatively modulates gene expression [20,21,22]. DNA methylation is catalyzed by enzyme-defined DNA methyltransferases (DNMTs) and, being it a reversible process, demethylation is catalyzed by the ten-eleven translocation (TET) enzymes. TET proteins are Fe^2+^-α-ketoglutarate dependent dioxygenases, and they catalyze an iterative oxidation reaction: acting on cytosines, TET proteins convert 5-methylcytosine to 5-hydroxymethylcytosine (5hmC), 5hmC to 5-formylcytosine (5fC), and 5fC to 5-carboxylcytosine (5caC) [23]. 5hmC is the first and most stable intermediate in the demethylation process and mainly localizes at the proximity of transcription factors binding sites, including distal regulatory elements, and in the body of highly expressed genes, suggesting a role in favoring gene expression regulation, inducing a loose chromatin structure, hence facilitating promoters and gene accessibility [24,25]. Analogously, histones can be subjected to epigenetic post translational modifications (PTMs). All core histone proteins contain intrinsically disordered N-terminal tails protruding from the DNA-wrapped region: they are noteworthy as target of enzymes whose aim is the addition or removal of chemical groups, comprehending—but not limited to—histone methyltransferases (HMTs) and histone acetyltransferases (HACs). Overall, by acting on the electrical charges of amino acids residues, PTMs prompt chromatin reshaping via downstream signaling: the so-called “histone code” hypothesis suggests a combinatory effect of different histone modification in orchestrating the epigenetic landscape of cells, modulating the underlying DNA activity and maintaining the genome stability, in the cell itself and between generations [26,27]. Out of the various histone epigenetic modifiers, the Polycomb group (PcG) proteins have gained much attention for their key role in epigenetic silencing for their involvement in cellular identity maintenance and as transcriptional regulators. In detail, enhancer of zeste homolog 2 (EZH2) acts as histone-lysine N-methyltransferase with a key role in gene repression by trimethylation of lysine 27 of histone H3 (H3K27me3). Similarly, other histone PTMs are associated with transcriptional silencing, such as trimethylation of lysine 9 on histone H3 (H3K9me3); on the contrary, several epigenetic markers are instructive for gene expression, for instance acetylation of lysine 9 on histone H3 (H3K9Ac) [28,29,30,31,32,33].

Methylation and demethylation are frequently observed to be deregulated in various diseases and cancer types, due to their importance in gene expression regulation. DNA methylation can change metabolism toward the development of multiple chronic syndromes [34]. Peroxisome proliferator-activated receptor alpha (PPAR-α) is a nuclear receptor expressed in active metabolic tissues such as liver, heart, brown fat, and skeletal muscle, where it regulates the expression of genes involved in lipid metabolism. A positive correlation has been found between the PPARA (gene encoding PPAR-α) DNA methylation level and the metabolic syndrome index, triglyceride concentrations and HOMA-IR (HOmeostatic Model Assessment for Insulin Resistance) [35]. Furthermore, adipose tissue inflammation, which is an important factor in the development of obesity and metabolic syndrome, has been associated with decreased DNA methylation of the gene encoding tumor necrosis factor (TNF) in subjects with metabolic syndrome compared to healthy subjects [36]. Indeed, TNF and many other inflammatory mediators are commonly found to be upregulated in insulin resistance and obesity [37]. Given the crucial role played by chromatin organization in modulating gene expression regulation, elucidating its architectural characteristics is of critical importance to understand genome function in health and disease. Therefore, this research aimed at investigating DNA methylation and demethylation, histones post-translational modifications, and the influence of epigenetics on chromatin organization under the effects of oxidative stress, which is notably implicated in several diseases, aging, and carcinogenesis.

## 2. Results

### 2.1. DNA Methylation and Demethylation Pattern Alteration following KBrO_3_ Treatment

**Figure 1:**
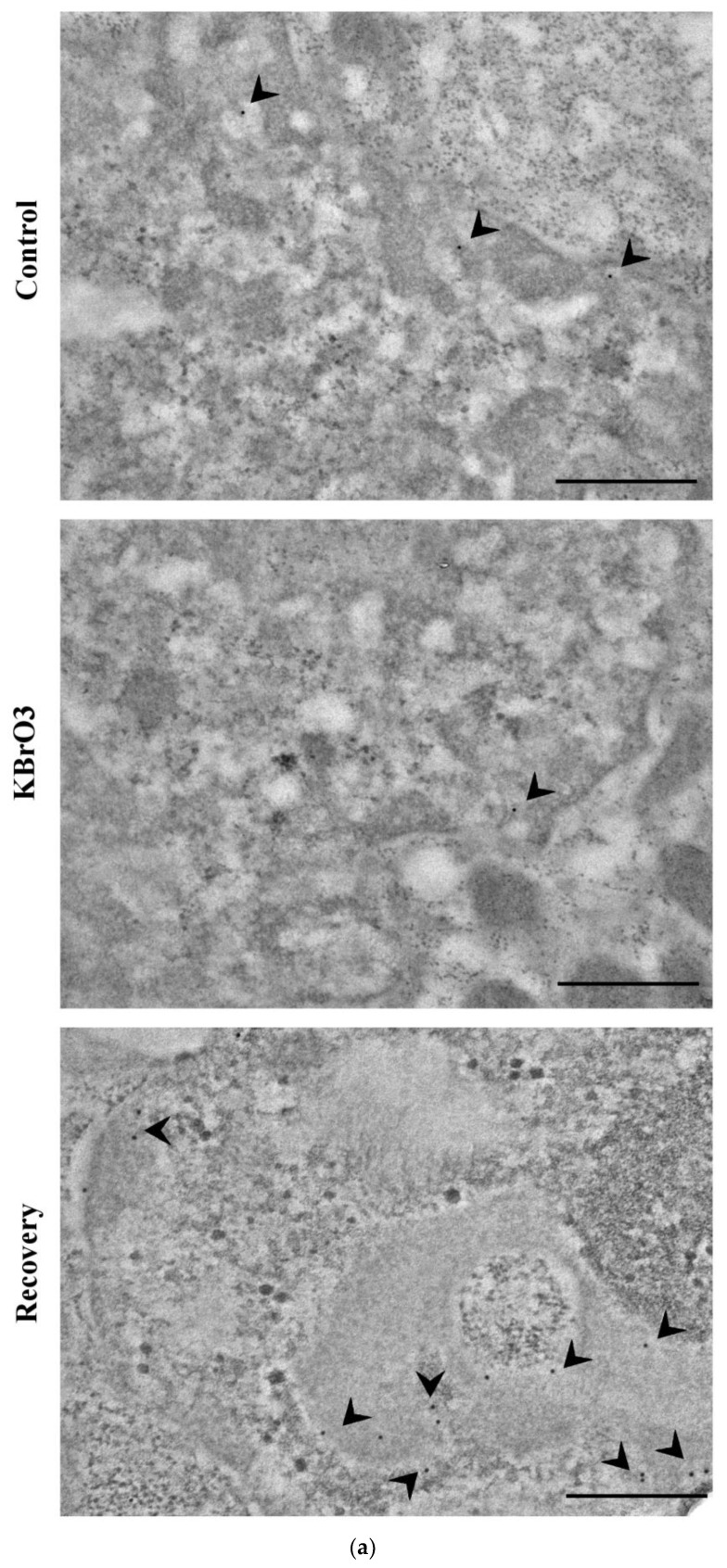

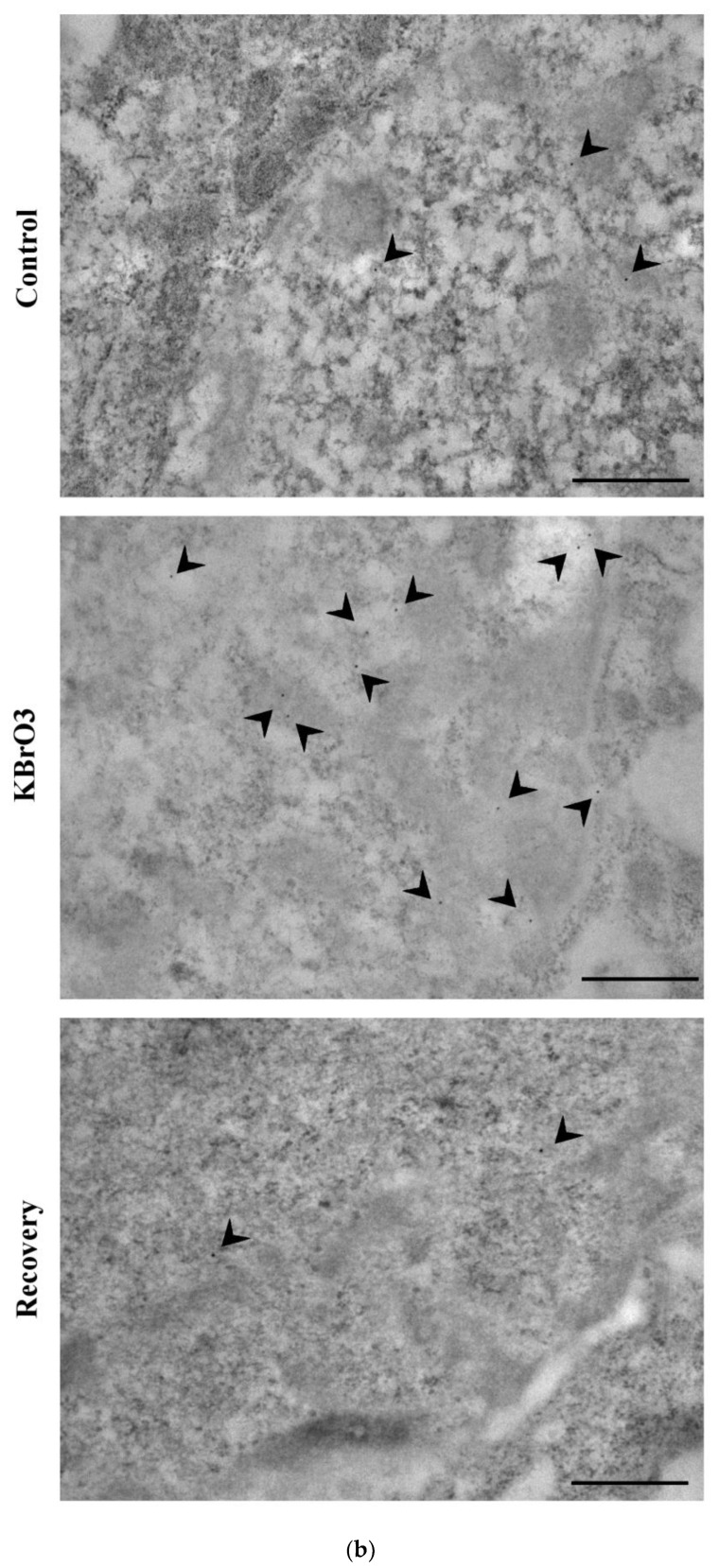

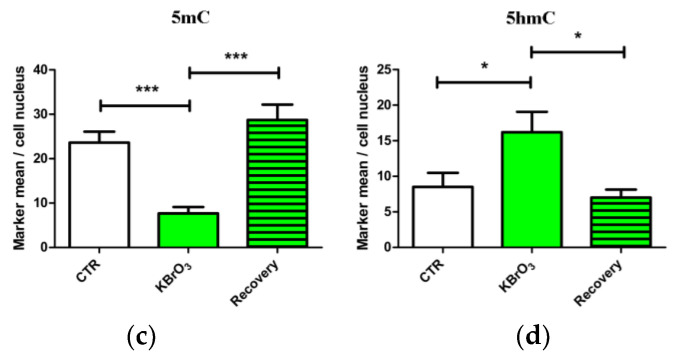
TEM micrograph showing (**a**) 5mC labelling (arrowheads) and (**b**) 5hmC labelling (arrowheads). Note 5mC localization in the EDTA-bleached heterochromatin and 5hmC main localization in the perichromatin region. Bars: 500 nm. (**c**,**d**) Histograms representing the abundancy of 5mC and 5hmC per nucleus. Asterisks identify colloidal gold counting-revealed significant differences. Mean with SEM are reported (*: *p* Value < 0.05; ***: *p* Value < 0.001).

Ultrastructural imaging followed by semi-quantitative analyses revealed significant alterations in the presence of 5-methylcytosine and in its oxidized form 5-hydroxymethylcytosine. Our results highlight a significant reduction in the presence of 5mC. On the contrary, we report an increment in 5hmC abundancy (Figure 1).

**Figure 2:**
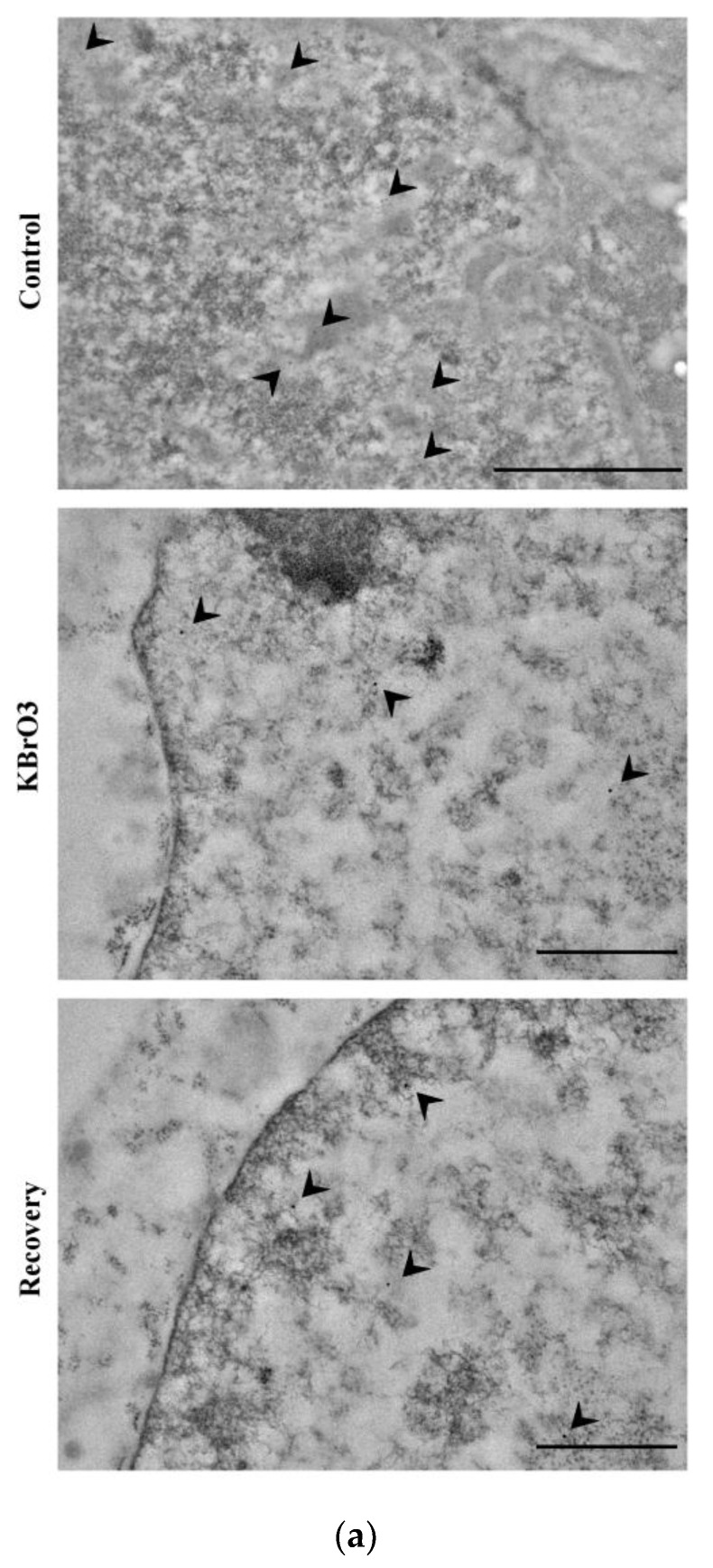

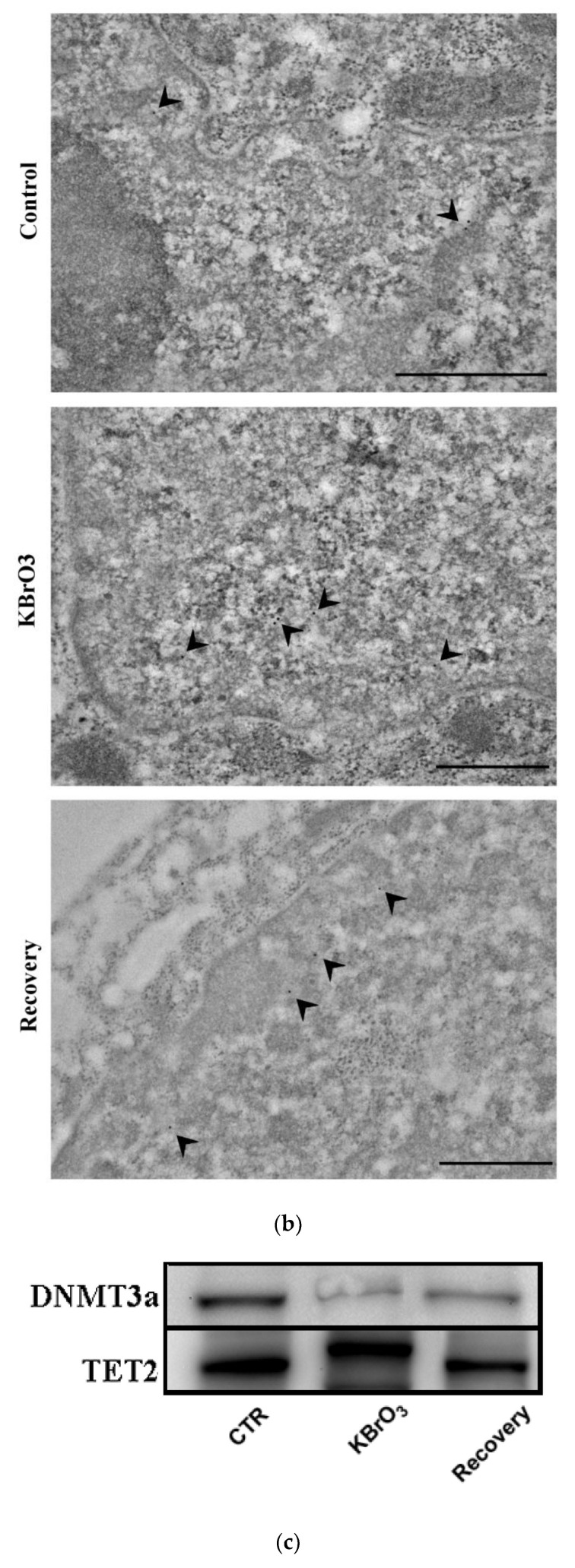

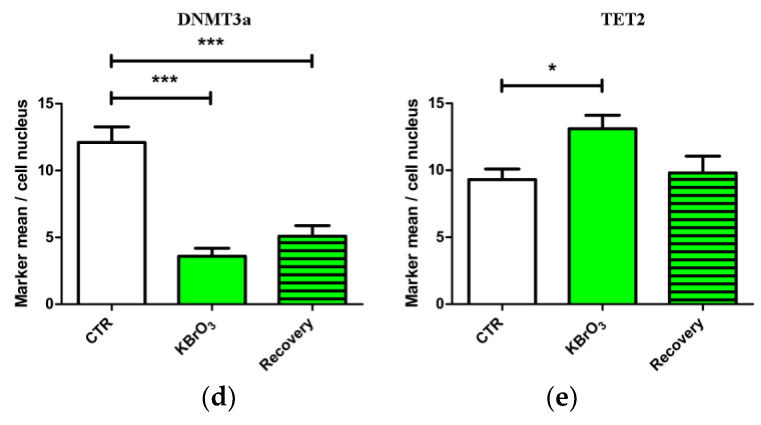
(**a**) DNMT3a labelling (arrowheads). The enzyme localized in the heterochromatic regions and at its surface. Bars: 1 μm (control); 500 nm (KBrO_3_ and recovery). (**b**) TET2 labelling (arrowheads). Note the localization at euchromatin-heterochromatin surface. Bars: 500 nm (control and KBrO_3_); 1 μm (recovery). (**c**) Western blot analysis of potassium bromate-treated cells with antibody targeting DNMT3a and TET2 (see Figure S1). (**d**,**e**) Histograms representing the abundancy of DNMT3a and TET2 per nucleus. Asterisks identify colloidal gold counting-revealed significant differences. Mean with SEM are reported (*: *p* Value < 0.05; ***: *p* Value < 0.001).

Coherently with 5mC reduction, our data show that KBrO_3_ treatment significantly reduces DNA methyltransferase 3a (DNMT3a) presence and lower values are also maintained in the recovery phase. Consistently with the reported increment in 5hmC abundancy, our analysis indicates a significant increase in TET2 levels (Figure 2).

### 2.2. The Histone Code Reflects Cell Transcriptional Activity

Then, we examined the distribution and performed statistical analyses assessing transcriptionally permissive or repressive histone markers: acetylated H3K9, tri-methylated H3K9, and tri-methylated H3K27, respectively.

**Figure 3:**
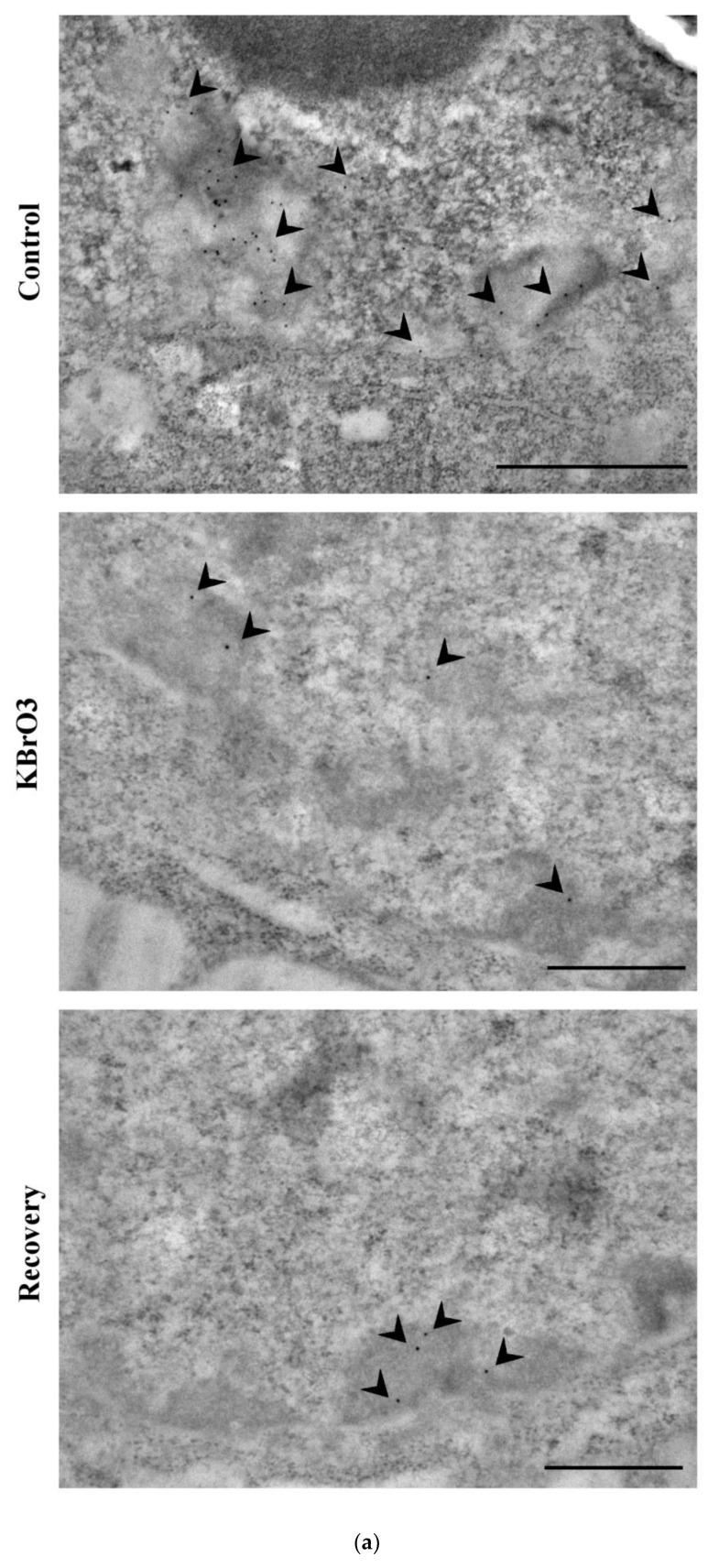

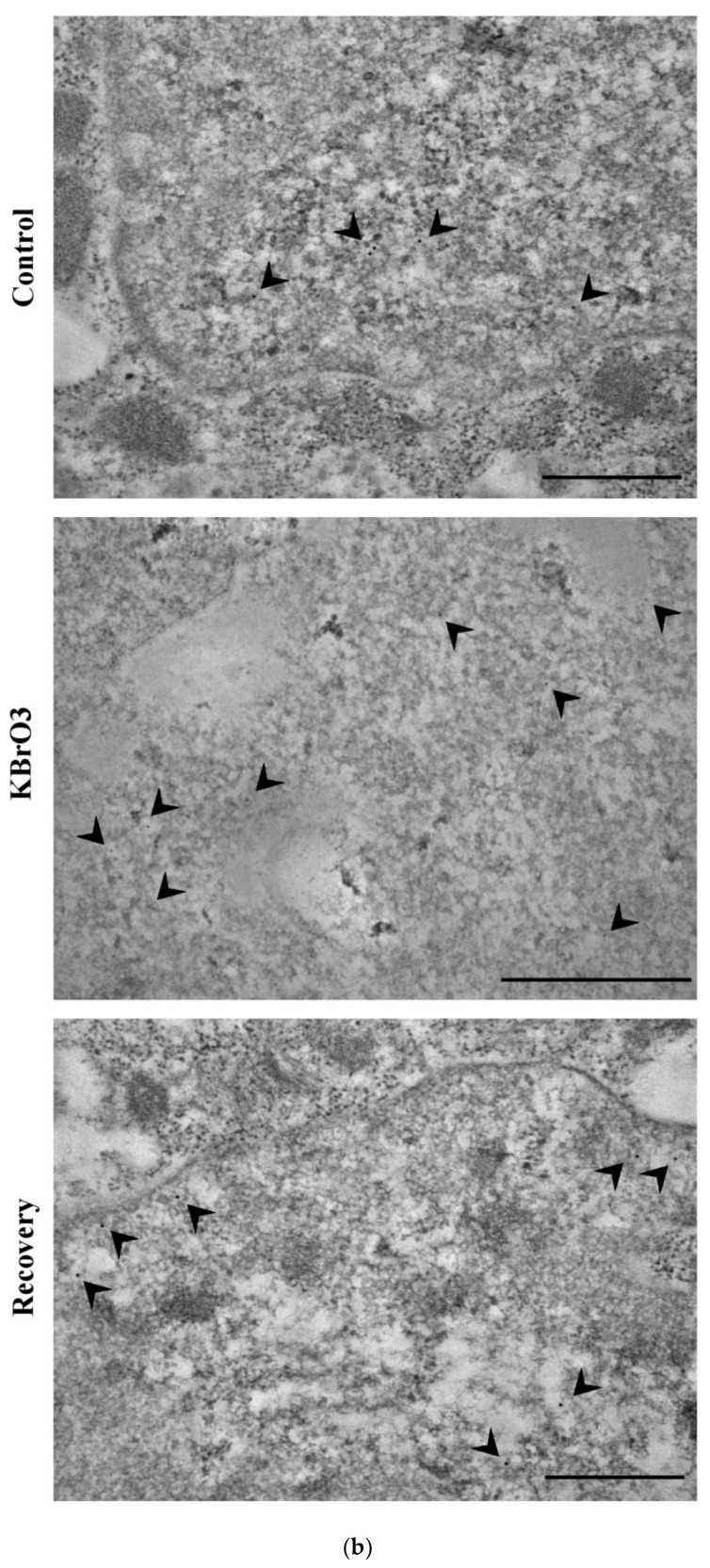

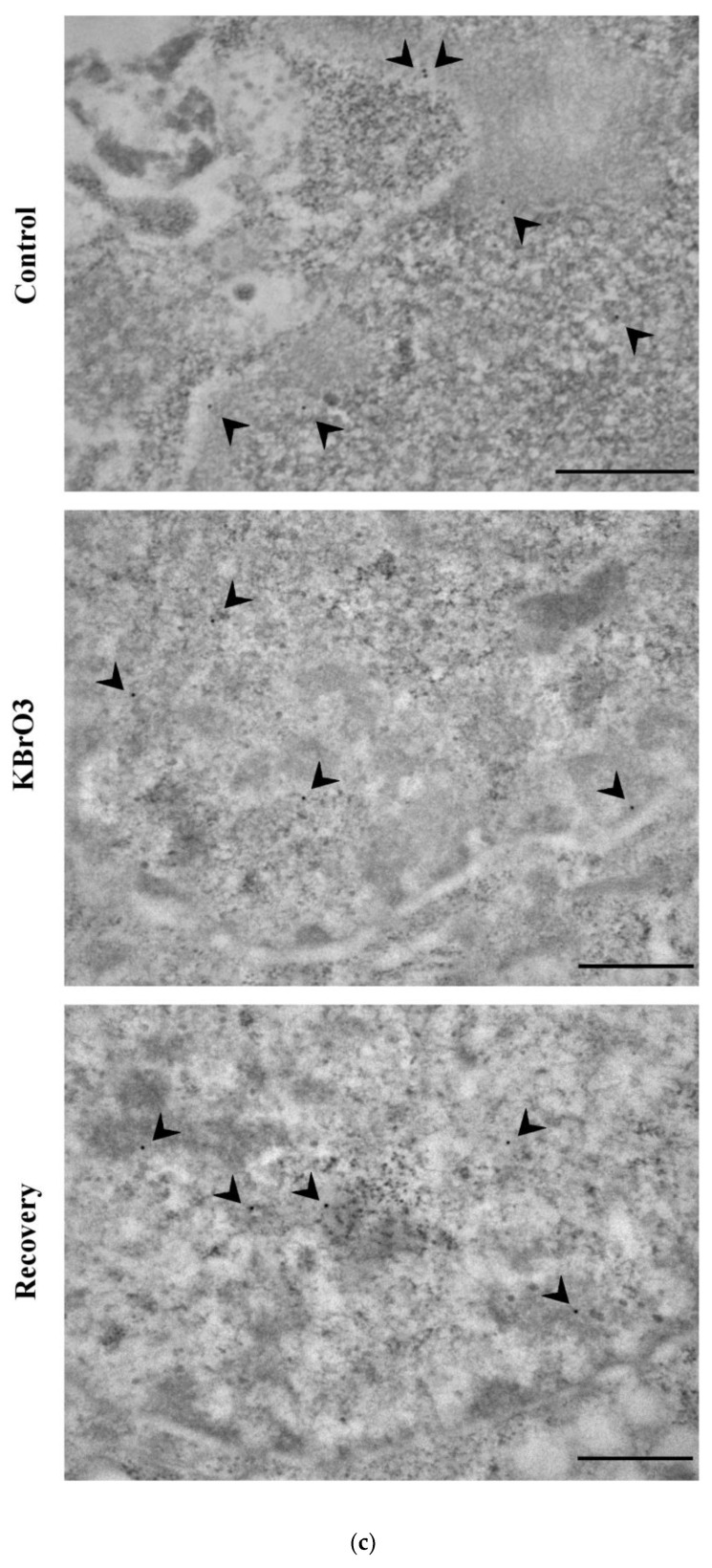

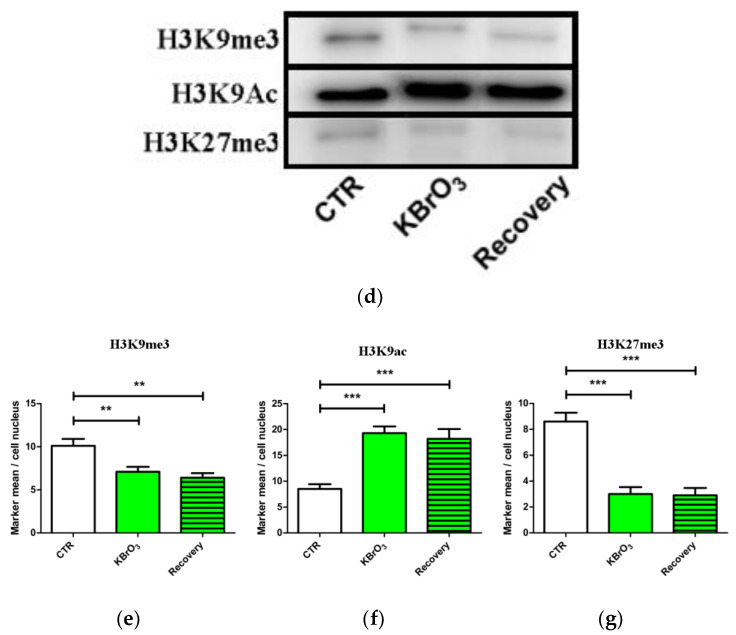
(**a**) H3K9me3 labelling (arrowheads). Note its localization in the heterochromatin. Bars: 1 μm (control); 500 nm (KBrO_3_ and recovery). (**b**) H3K9Ac labelling (arrowheads). It localizes in the euchromatin and in the perichromatin region. Bars: 500 nm (control and recovery); 1 μm (KBrO_3_). (**c**) H3K27me3 (arrowheads). This histone-PTM is found in the three already highlighted chromatin compartments. Bars: 500 nm. (**d**) Western blot analysis of potassium bromate-treated cells with antibody targeting H3K9me3, H3K9Ac and H3K27me3 (see Figure S1). (**e**–**g**) Histograms representing the abundancy of the histone PTMs per nucleus. Asterisks identify colloidal gold counting-revealed significant differences. Mean with SEM are reported (**: *p* Value < 0.01; ***: *p* Value < 0.001).

Potassium bromate induces a statistically significant reduction in the amount of the repressive histone markers H3K9me3 and H3K27me3. On the contrary, our data show that the treatment determines an increase in the transcription permissive histone modification H3K9Ac (Figure 3).

### 2.3. EZH2 Localization Is Altered following Oxidative Insult

Next, we focused our attention on EZH2, analyzing its expression and cellular localization by combining qPCR and super-resolution microscopy. 

**Figure 4:**
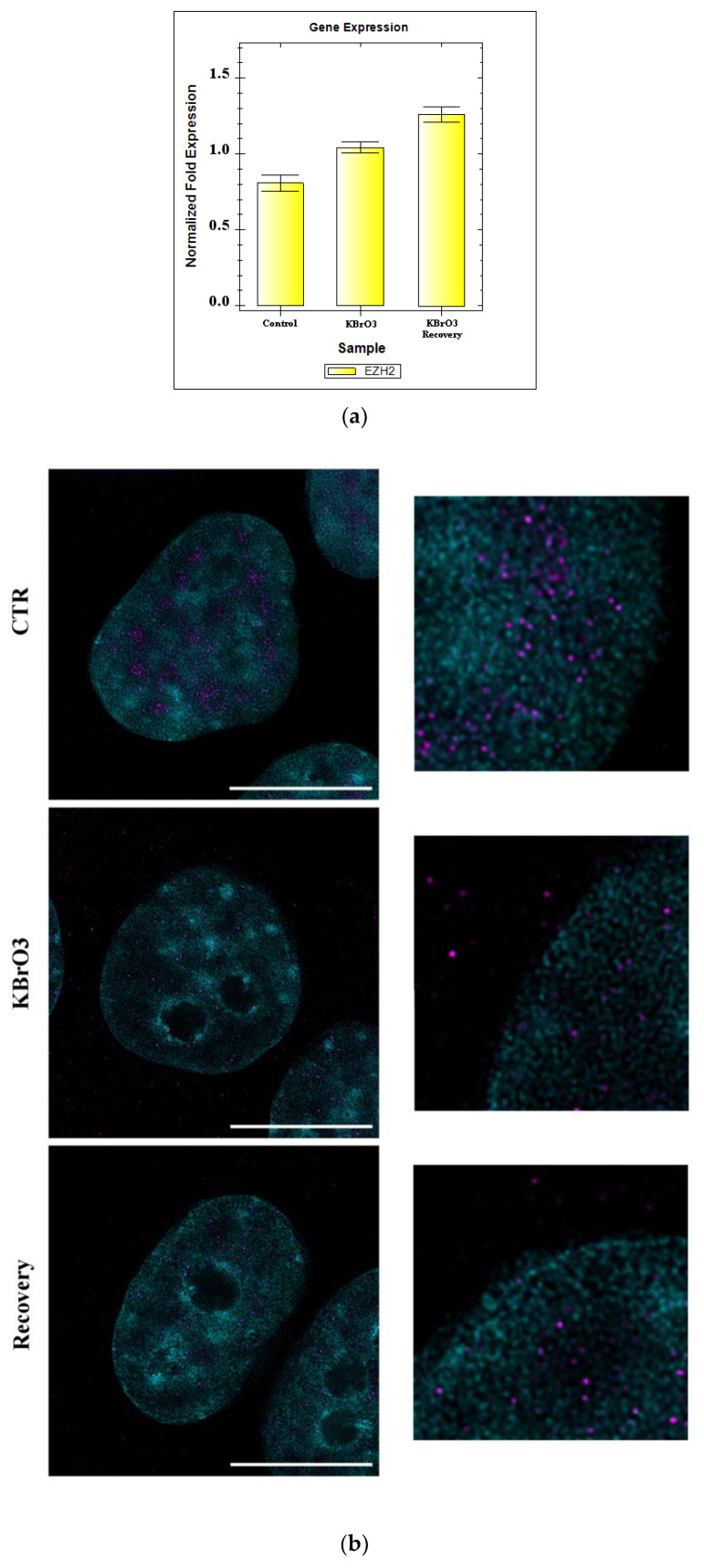

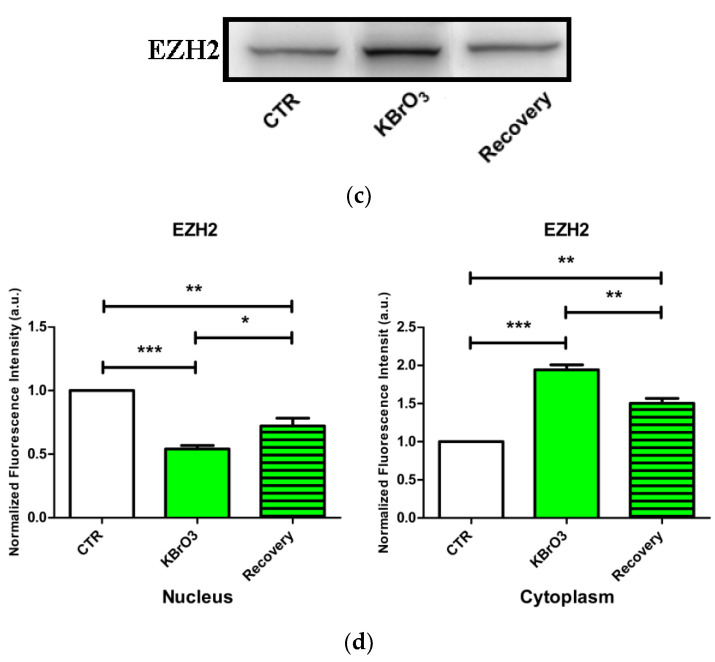
(**a**) qPCR gene expression analysis. No statistical significance differences are highlighted. (**b**) Dual STED displaying DNA (cyan) and EZH2 (magenta) in HeLa cells during experimental conditions. Note the diverse EZH2 signal in each condition. Bars: 10 μm. (**c**) Western blot analysis of potassium bromate-treated cells with antibody targeting EZH2 (see Figure S1). (**d**) Histograms representing the normalized fluorescence intensity of EZH2 signal in the nucleus and in the cytoplasm. Note the nuclear labelling reduction and the simultaneous cytoplasm signal increment during KBrO_3_ stress. Asterisks identify statistically significant differences (*: *p* Value < 0.05; **: *p* Value < 0.01; ***: *p* Value < 0.001).

Since qPCR indicates that EZH2 mRNA abundancy is not directly affected by the experimental conditions, we investigated whether the protein localization may be altered. Dual stimulated emission depletion (STED) microscopy observations allowed precise EZH2 protein localization as well as structural analysis of the chromatin organization. Analysis of untreated cells shows that EZH2 preferentially localizes at the borders of heterochromatin and in nuclear regions mostly devoid of chromatin. When assessing the oxidative stress condition, a slight decrease in EZH2 nuclear abundancy is revealed; the signal also appears mostly diffused in the nucleoplasm compared to the spot-like signal condition typical of untreated cells. However, differently from the control, EZH2 is also clearly visible in the cytoplasm, that is verified also during the recovery (Figure 4).

### 2.4. Oxidative Stress and Chromatin Reorganization

Then, we investigated the effects of KBrO_3_-induced oxidative stress on chromatin organization. First, transmission electron microscopy analysis highlighted the formation of several, small, condensed chromatin clusters. It is noteworthy to report that these data confirm the previously reported results obtained with STED microscopy. At the same time, observation of the cytoplasm did not highlight severely compromised organelles, as their ultrastructure is left mainly unaltered, hence supporting data according to which KBrO_3_ does not alter the cellular microenvironment.

**Figure 5:**
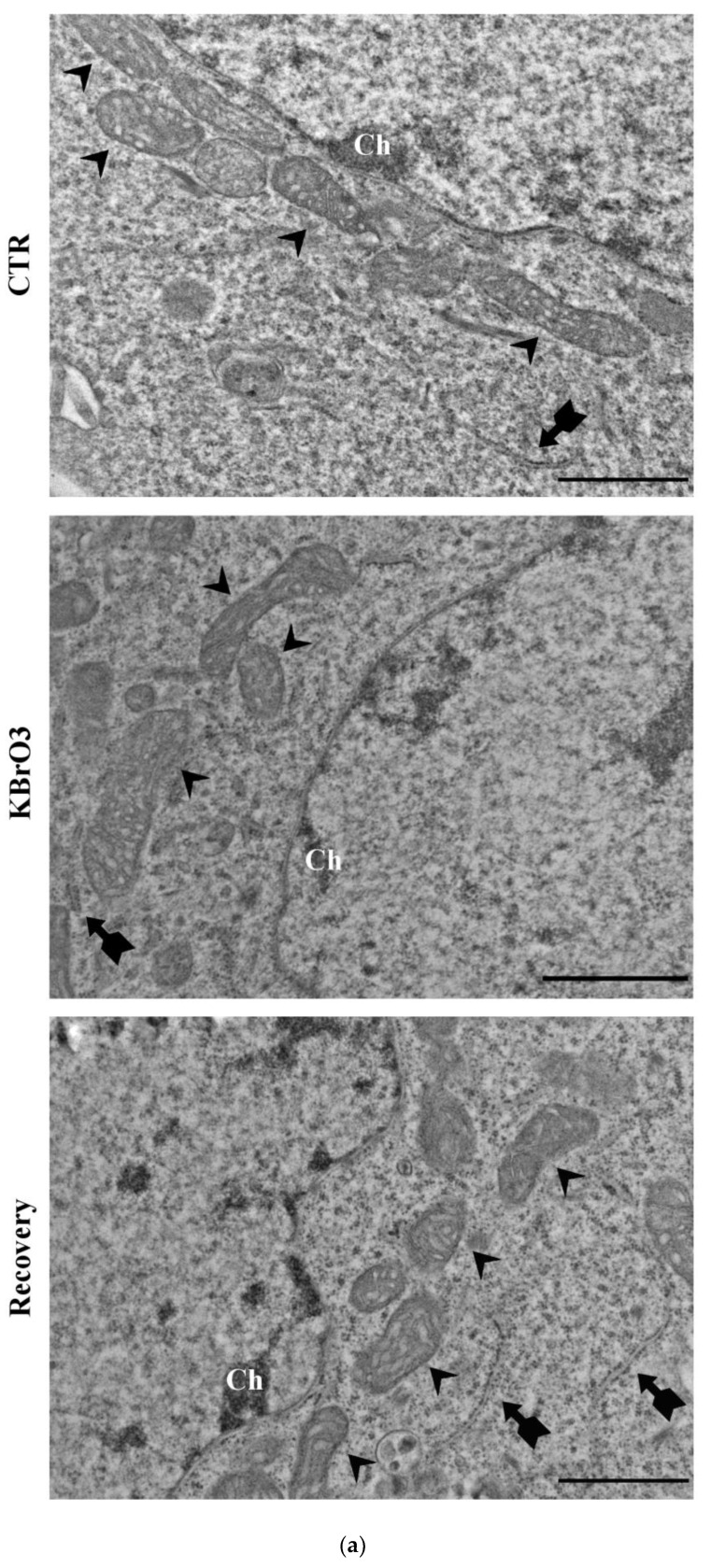

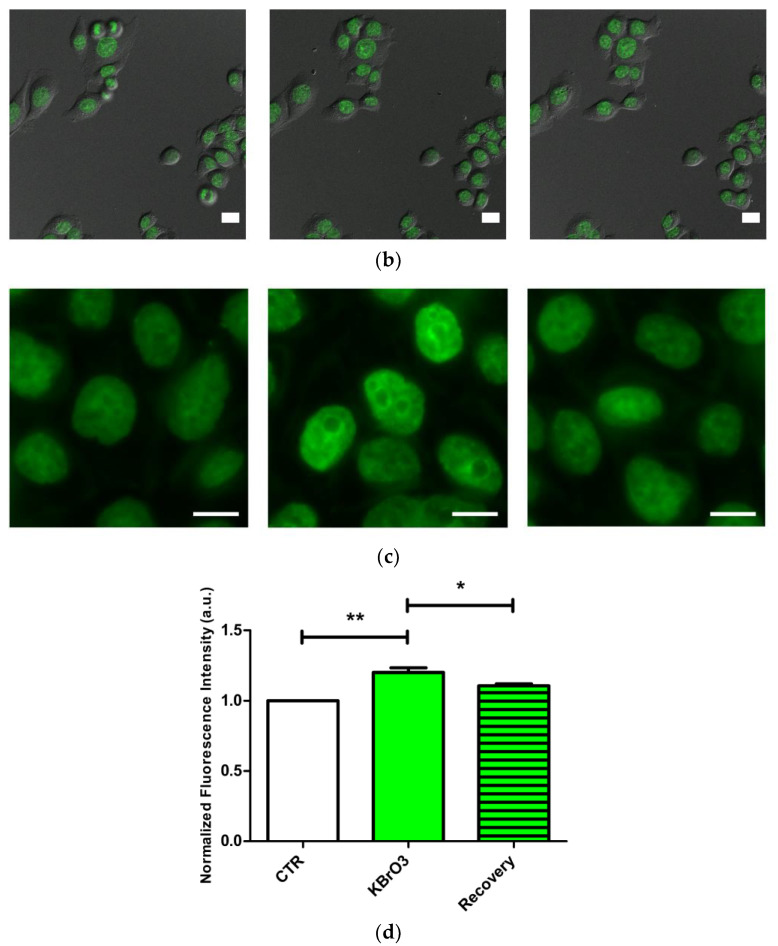
(**a**) Ultrastructural analysis of cell treated with potassium bromate. KBrO_3_ does not alter the subcellular environment: note the active mitochondria morphology (arrowheads), the unaltered RER and ribosomes (flagged arrows); few heterochromatic areas (Ch) are visible in the nucleus. Bars: 1 μm. (**b**) Timelapse frames (pre-, during and post-treatment respectively) showing rapid chromatin reorganization in clusters in cells subjected to KBrO_3_ oxidative insult. Bars: 10 μm. (**c**) Note the difference in chromatin fluorescence between pre-KBrO_3_ treatment (**left panel**), during the oxidative stress (**middle panel**) and during the recovery phase (**right panel**). Bars: 10 μm. (**d**) The histogram shows the significant KBrO_3_-induced increment of the nuclear fluorescent signal, indicative of the formation of signal-positive condensed chromatin cluster (*: *p* Value < 0.05; **: *p* Value < 0.01).

Based on the chromatin rearrangement, live cell imaging has been performed to monitor the discussed chromatin reorganization step-by-step. The timelapse displays the gradual chromatin condensation in clusters during the experimental condition. Notably, after removal of the oxidizing agent, the nuclear compartment reverts to the initial organization (Figure 5).

### 2.5. Oxidative Stress Induction Assessment

**Figure 6:**
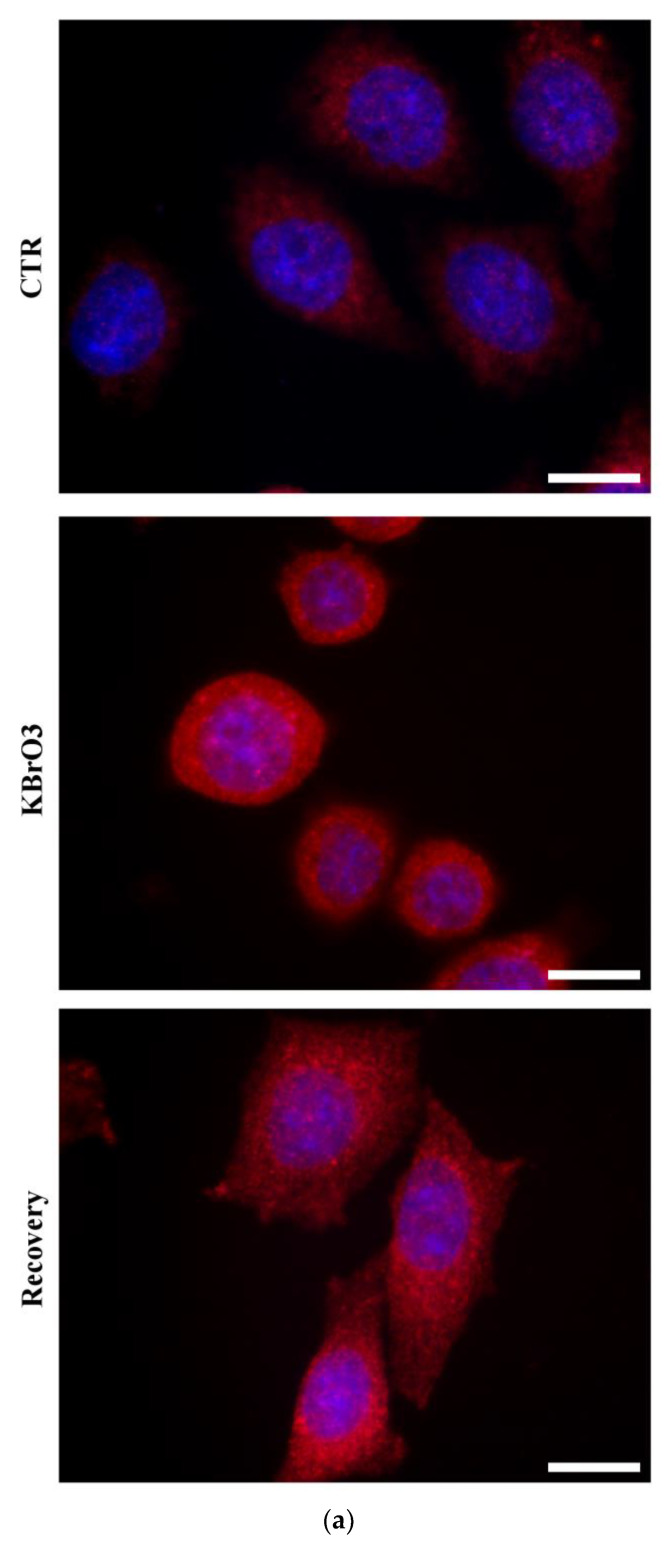

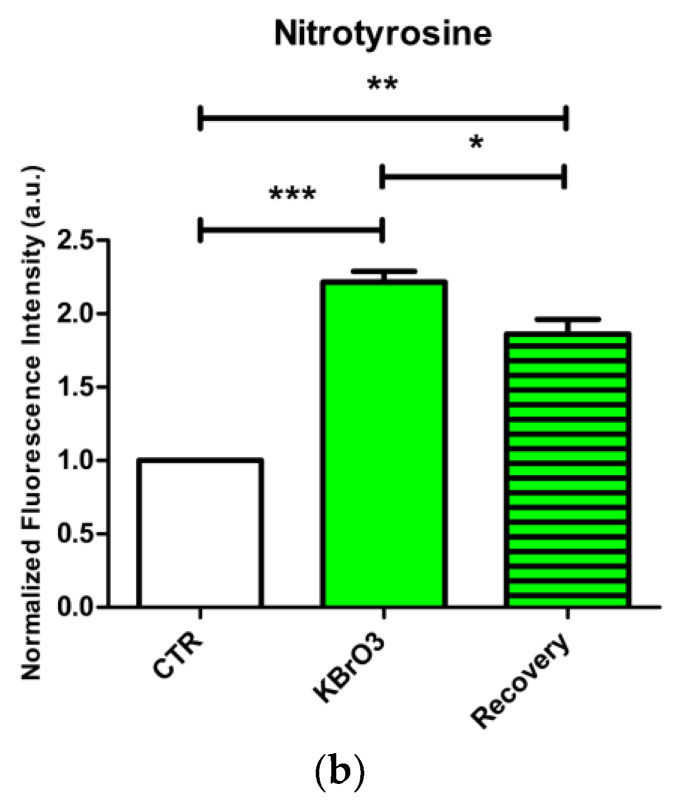
(**a**) Widefield fluorescence displaying DNA (blue) and nitrotyrosine (red) in HeLa cells during experimental conditions. Note the significant signal intensity increment during KBrO_3_ treatment. Bars: 10 μm. (**b**) Histogram representing the normalized fluorescence intensity of nitrotyrosine signal. Asterisks identify statistically significant differences (*: *p* Value < 0.05; **: *p* Value < 0.01; ***: *p* Value < 0.001).

As further confirmation about the efficacy of the approach, we tested the efficiency of the KBrO_3_ treatment by measuring cellular nitrotyrosine level as biomarker of oxidative stress. Widefield fluorescence quantitative analysis highlighted a significant increment in the nitrotyrosine labelling during the potassium bromate treatment compared to the control and recovery conditions (Figure 6). This relative measurement confirms the effectiveness of the KBrO_3_ as oxidative stress inducer.

## 3. Discussion

In this work we assessed the effect of KBrO_3_-induced oxidative stress on nuclear epigenetic phenomena and chromatin modifications. We selected HeLa cells for their high metabolic rate, in order to maximize the response at the level of gene expression modulation; moreover, owing to its high expression profile, this model could partially mimic accessible chromatin dynamics already identified during endocrine pancreas development and several other endocrine cells differentiation [38,39].

Taken together, our data demonstrate that KBrO_3_ treatment-induced oxidative stress profoundly alters chromatin organization and several epigenetic patterns in cell nuclei. After evaluating treatment efficiency measuring ROS level by nitrotyrosine assessment, a promising biomarker for oxidative stress [40,41], we have shown significant reductions in 5mC and in its corresponding DNA-targeting enzyme DNMT3a. Moreover, to better understand epigenetic regulation of chromatin structure, we investigated histone PTMs during the experimental conditions, considering both PTMs instructive for gene silencing (H3K9me3 and H3K27me3) and transcription (H3K9Ac). Coherently with data obtained from DNA methylation analysis, statistically significant drops in the histone markers for silenced chromatin regions H3K9me3 and H3K27me3 have been described. On the contrary, a raise in H3K9Ac levels has been identified, further confirming the idea that KBrO_3_ induces a positive gene expression epigenetic profile. Finally, live cell imaging allowed the progressive observation of small heterochromatin clumps formation during the treatment, as well as their dissolving in the recovery, suggesting chromatin rearrangement for specific silencing and activation of genes according to their function during this source of stress. Paired with dual STED approach, which associates analyses conducted at the level of DNA with findings about Polycomb Repressive Complex 2 catalytic subunit EZH2, we revealed a characteristic nuclear distribution of EZH2, which mainly localizes in areas devoid of heterochromatin clumps and at the borders of these clusters, with a significant reduction of its level and a contemporary increment in the cytoplasmic signal during the stress condition. While our data clearly identify a form of EZH2 migration from the cell nucleus to the cytoplasm, qPCR does not highlight statistical differences in its gene expression level. We hypothesize this discrepancy to be due to the experimental parameters and the specificity of KBrO_3_ treatment as not excessively altering the cellular microenvironment. Indeed, Polycomb Repressive Complexes have already been identified as involved in epigenetic response to certain type of stress [42,43], hence we could speculate that by increasing the exposure time or concentration of potassium bromate a rapid increment in EZH2 would be detected also by qPCR. Remarkably, our experiment shows a trend consisting in a constant gene expression increment, almost reaching significant values the longer the time point advanced from the stress source exposure. Since other groups reported similar expression parameters and, notably, a cytoplasmic EZH2 relocalization [44,45], it is tempting to speculate a constant increment in EZH2 mRNA abundancy (as already identified by microscopy and western blot quantifications at the protein level), but further studies are required to clarify this question. Together with the findings from these groups, our data suggest a main role of EZH2 and of the Polycomb-group family in reshaping the nuclear architecture and in gene expression regulation during the experimental conditions.

Our data agree with numerous other findings. First, our results confirmed extensive chromatin reorganization under oxidative stress conditions, as described by Madugundu and colleagues, validating the observation of a specific increment in 5mC oxidation products [46]. Moreover, our data are also in accordance with Kietzman and colleagues, who described the importance of ROS in altering the epigenetic landscape, affecting DNA methylation, post-translational histone modifications, and non-coding RNA transcripts and their effect on cardiovascular diseases [47]. Furthermore, the chromatin rearrangement following oxidative stress shown here is in agreement with various works [48,49], that reported profound nuclear reorganization caused by oxidative stress in neurodegenerative models. ROS effect on histones represents another important topic: since histones are the most abundant chromatin proteins, any change in their PTMs have a critical outcome on chromatin structure, hence influencing genome stability and gene expression [50]. We linked KBrO_3_ effect with a general reduction in repressive PTMs, while transcription-instructive ones are statistically increased. Coherently, a similar gene expression upregulation scenario was described by García-Giménez and colleagues, although they focused their attention on histone H3 glutathionylation only [51]. Furthermore, multiple studies described a putative role for ROS as enhancers of histone deacetylases activity [52,53]; nonetheless, this aspect is still largely debated [50]. Thus, this work integrated in a series of research activities highlights the relevance-gaining topic of chromatin reorganization after stress stimuli: preservation of genome stability and gene expression regulation are essential features concerning significant issues such as the way cells respond to damage, pathophysiological conditions, or aging [54,55].

The process of mRNA transcription constitutes a critical step of the gene flow information and employs multiple essential regulators to produce a faultless cellular proteome to maintain healthy physiology of the whole organism. Therefore, it is not surprising that any abnormalities, caused by external or internal insults, may contribute to a perturbed homeostasis and consequently human diseases. Acute oxidative stress is one of the major causes of such imbalances and it is known to induce epigenetic changes and dysregulation of gene expression in pathological cases and in ageing [56,57]. For instance, obesity has gained huge importance in the past decades as the number of people with condition is constantly rising. Obesity holds its roots in a complex lifestyle–gene interaction, deeply interconnected to epigenetic alterations. Since preventive measures are often reported to fail, interest in new approaches is rising, for example in the field of nutraceuticals using antioxidants to overcome acute oxidative stress [58,59]. Similarly, ROS are key players in development of low-grade inflammation, which in turn is involved in the physiopathology of polycystic ovary syndrome (related to insulin-resistance, immune dyscrasia and much more), as well as in metabolic syndrome-related manifestation, as atherosclerosis, hypertension, and T2D: hence, a profound understanding of the link between onset of such conditions and their genetic bases (for instance, at the level of the histone code and overall chromatin organization) could be reflected in discovery of new targets and development of new therapies aiming at ameliorating patients’ life conditions [60,61]. Inhibiting oxidative damage may have therapeutic benefits, thus administration of vitamins, polyphenols, and antioxidant molecules is reasonably reported to have ameliorating effect on patients [62,63,64,65]. Moreover, several epigenetic drugs are already being tested and in the near future their combination with antioxidants treatments may pave the way for novel possibilities [66,67,68]. For instance, in the recent years interest in chronic oxidative stress is rising, motivated by the modern focus on space technology and transportation. Of particular interest will be the impact on the health of astronauts: combining basic data from research in genetic and medical fields with validated antioxidant-integrated dietary regime could be a critical [69,70,71]. Integration of endocrine tissue-, gene-, and oxidative stress-specific studies of epigenetic modifications will deepen the knowledge and strengthen the therapeutical approaches, positively impacting drug design and therapies to ameliorate conditions and increase the lifespan of patients and people with high risk of endocrine diseases [72,73].

## 4. Materials and Methods

### 4.1. Cell Culture and Treatment

HeLa cells were cultured in Dulbecco’s modified Eagle medium (DMEM) supplemented with 10% fetal bovine serum (FBS) and 1% penicillin/streptomycin at 37 °C in a humidified atmosphere (95% air/5% CO_2_).

48 h before experiments, cells were grown in 25 cm^2^ flasks. At approximately 70% confluence, cells were subjected to potassium bromate treatment by replacing the culture medium with fresh medium containing 40 mM KBrO_3_ for 1 h at 37 °C; for the recovery condition, after the treatment cells were allowed to recover in fresh medium for 1 h at 37 °C [16].

### 4.2. Transmission Electron Microscopy Sample Preparation

Control and treated cells were harvested by mild trypsinization with 0.25% trypsin in PBS containing 0.05% ethylene diamine tetraacetic acid (EDTA), centrifuged at 800 rpm for 5′ and then fixed for morphological analysis or for immunocytochemistry.

For immunocytochemistry, cells were fixed with 4% paraformaldehyde (PFA) in culture medium for 2 h at 4 °C. The cell pellet was rinsed three times in PBS for 10′ each, pre-embedded in 2% agarose in water, incubated in NH_4_Cl 0.5 M for 30′ and dehydrated in graded ethanol (2 × 5′ 30%, 2 × 10′ 50%, 2 × 10′ 70%, 2 × 15′ 90%, 3 × 10′ 100%) and then embedded in acrylic resin (LR White, Agar Scientific, Stansted, UK).

For morphological analysis, cells were fixed with 2.5% glutaraldehyde in culture medium for 2 h at room temperature (RT). The cell pellet was rinsed three times in PBS for 10′ each, post-fixed in 1% aqueous OsO_4_ for 2 h at room temperature and rinsed three times in H_2_O for 10′ each. Cells were pre-embedded in 2% agarose in water and dehydrated in graded acetone (2 × 5′ 30%, 2 × 10′ 50%, 2 × 10′ 70%, 2 × 15′ 90%, 3 × 10′ 100%) and then embedded in epoxy resin (EM-bed812, Electron Microscopy Sciences, Hatfield, PA, USA).

In both cases, ultrathin sections (60–80 nm) were cut on a Reichert OM-U3 ultramicrotome and collected on nickel grids (200 Mesh). The specimens were observed with a JEM 1200 EX II (JEOL, Peabody, MA, USA) electron microscope operating at 100 kV and equipped with a MegaView G2 CCD camera (Olympus OSIS, Tokyo, Japan).

#### Transmission Electron Microscopy Immunocytochemistry and Sample Staining

Grids for immunocytochemical analyses were first incubated in a drop of normal goat serum (NGS) diluted in PBS and then incubated over-night (ON) in a drop of primary antibody (Table 1). Then, grids were rinsed twice in PBS/TWEEN20 for 5′ and twice in PBS for 5′, incubated in NGS and finally in the proper colloidal gold-particle conjugated-secondary antibodies for 30′ at RT. Grids were rinsed twice in PBS for 5′ and twice in dH_2_O for 5′.

Staining for immunocytochemistry was performed according to the EDTA regressive technique [74]. Grids were incubated in 4% uranyl acetate solution at RT for 2 min, followed by a 30 s treatment in 0.2 M EDTA at RT. Then, grids are incubated in the lead citrate solution for 2 min at RT. Every step is followed by rapid rinsing in dH_2_O.

Staining for morphological analysis was achieved via uranyl acetate and lead citrate staining [75]. Grids were incubated in a 4% uranyl acetate solution at room temperature (RT) for 10 min. Then, several rinses were rapidly performed in dH*_2_*O. Subsequently, grids were incubated in lead citrate at RT for 2 min, followed by rapid rinses in dH_2_O.

### 4.3. Widefield and Confocal Microscopy Sample Preparation and Immunocytochemistry

Treated and control HeLa cells were grown on coverslips and fixed with 4% formalin for 20′ at RT. Cells were then post-fixed with 70% ethanol at −20 °C for at least 24 h. Samples were rehydrated for 10′ in PBS at RT and processed for DNA staining or immunocytochemistry. DNA was stained with either 0.1 μg mL^−1^ Hoechst 33258 for 5′, followed by two rinses in PBS of 5′ each or SPY505-DNA 20X (Spirochrome, Stein am Rhein, Switzerland) followed by a quick rinse in dH_2_O. For immunocytochemistry, cells were incubated in the blocking buffer for 20′ and then in the primary antibody (Table 2) for 1 h at RT. Then, after three washes in PBS of 5′ each, samples were incubated with the proper secondary antibody for 45′ at RT. After 5 washes in PBS of 5′ each, DNA was stained with Hoechst 33258 or SPY505-DNA 20X as already described. Coverslips were mounted using Mowiol and observed with a Leica TCS SP8 STED 3X (Wetzlar, Germany).

### 4.4. Live Cell Imaging

HeLa cells were grown on coverslip and stained with SPY505-DNA 1X in complete medium. After incubating 1 h at 37 °C, cells were observed with a Leica TCS SP8 STED 3X equipped with the incubation chamber for in vivo imaging. Cells were treated for potassium bromate stress and for the correspondent recovery condition as previously indicated.

### 4.5. Stimulated Emission Depletion Microscopy Sample Preparation and Immunocytochemistry

Treated and control HeLa cells were grown and processed as already described for confocal microscopy. Samples were observed with a Leica TCS SP8 STED 3X equipped with a 660 nm depletion laser.

### 4.6. Western Blotting

Treated and control cells were detached by mild trypsinization and centrifuged at 180 g for 10 min and rinsed twice in PBS. Then, samples were incubated in Radioimmunoprecipitation assay (RIPA) lysis buffer 2X (Nonidet P-40 2% (Sigma-Aldrich, St. Louis, MO, USA) Deoxycholic acid 1% (Sigma-Aldrich), sodium dodecyl sulphate (SDS) 0.2% (SERVA Electrophoresis GmbH, Heidelberg, Germany) in PBS pH 7.4) with addition of protease and phosphatase inhibitors (aprotinin 100 µg/mL (Sigma-Aldrich), leupeptin 200 µg/mL (Sigma-Aldrich), PMSF 4 mM (Sigma-Aldrich), Na_3_VO_4_ 4 mM (Sigma-Aldrich), NaF 4 mM (Sigma-Aldrich)). Proteins were quantified using Bicinchoninic acid (BCA) (Euroclone, Milano, Italy) assay and absorbance was measured at a wavelength of 562 nm using a double beam spectrophotometer SHIMADZU UV-1601 (Kyoto, Japan). Samples were dissociated using SDS 3X + 3% β-mercaptoethanol (Sigma-Aldrich).

Samples were boiled at 96 °C for 3 min, centrifuged at 18,000× *g* and then separated by SDS page in a discontinuous system composed of a stacking gel (3%) and a running gel (6%, 10%, and 15%) according to the proteins molecular weight. Precision Plus Protein Standards All Blue (Bio-Rad, Hercules, CA, USA) were loaded as ladder. Electrophoresis was performed in an electrophoretic chamber MINI-PROTEAN III (Bio-Rad) applying a constant voltage of 150 V for 60 and 120 min.

Proteins were transferred from the gel on a polyvinylidene difluoride (PVDF) (Bio-Rad) using an electrophoretic chamber MINI-PROTEAN III (Bio-Rad) applying a constant electric current of 200 mA for 2 h. After 60′ incubation in a solution of 5% BSA in Tris-buffered saline (TBS), PVDF was incubated in primary antibodies (see Table 1 and Table 2) diluted 1:1000 in BSA 5% in TBS, NaN3 0.02%, Tween20 0.1% ON at 4 °C. After five rinses in washing buffer (PBS + Tween20 0.1%) for 5′, PVDF was incubated with the proper horseradish-peroxidase-conjugated secondary antibody diluted 1:2000 (anti-rabbit) or 1:5000 (anti-mouse) in washing buffer for 60′ at RT. After rinsing in washing buffer, proteins were finally visualized using an enhanced chemiluminescence (ECL) kit and the light emission was detected using a ChemiDoc XRS (Bio-Rad).

### 4.7. Real Time PCR (qPCR)

Treated and control cells were detached by mild trypsinization and centrifuged at 800 rpm for 5′. RNA was extracted (RNeasy Kit, Qiagen, Hilden, Germany) and quantified using the Nanodrop spectrophotometer (Bio-Rad).

mRNA was retrotranscribed using the Superscript IV protocol (Invitrogen), heating at 65 °C for 5′ 50 ng/uL random examers, 10 mM dNTPs mix and template RNA in RNase-free water. After incubation on ice, the Superscript IV buffer, 100 mM dithiothreitol, ribonuclease inhibitor and the Superscript IV reverse transcriptase (200 U/Ul) were added and the thermal-cycler was run under the following parameters: 23 °C for 10′, 55 °C for 10′ and 80 °C for 10′. The analysis was conducted assessing EZH2 expression using RPS18 and GADPH as reference genes. Primers were selected using following Primer3 setup: 18-20-23 nucleotides length, 58-60-63 °C melting temperature with a maximum difference of 2 °C between forward and reverse primers, 100-300 product size range, resulting in finale 281 product size. EZH2 forward sequence (5′ to 3′): GCGGATAAAGACCCCACCAA; EZH2 reverse sequence (5′ to 3′): GCTGGGCCTGCTACTGTTAT. RPS18 forward sequence (5′ to 3′): ATTAAGGGTGTGGGCCGAAG; RPS18 reverse sequence (5′ to 3′): AAGTGACGCAGCCCTCTATG. GADPH forward sequence (5′ to 3′): CCACAGTCCATGCCATCACT; GADPH reverse sequence (5′ to 3′): GCCTGCTTCACCACCTTCTT. Data were analyzed using CFX Manager Software Version 1.5 (Bio-Rad).

### 4.8. Data and Statistical Analysis

Three independent replicates were performed for each experiment a statistical evaluation was conducted on. For EM immunolabelling, secondary antibody-conjugated colloidal gold particles were counted by at least two independent operators. Ten nuclei showing similarities in size, nuclear envelope extension, and in the ratio between heterochromatin and euchromatin were selected, in order to exclude that possible differences could be attributed to fundamental cell differences. One-way ANOVA with Bonferroni’s post hoc test was performed using GraphPad Prism version 5.03 (GraphPad Software, La Jolla, CA, USA) and mean with standard error of the mean (SEM) are reported in the plots. *p*-values from *p* < 0.05 were considered statistically significant.

Except when specifically indicated, images were processed using ImageJ2 and Fiji [76,77], according to the standard of ethics in image processing. Deconvolution was performed using the Huygens Software (Scientific Volume Imaging, Hilversum, The Netherlands, http://svi.nl accessed on 15 November 2022). Tables were assembled using Jasc Paint Shop Pro version 7.02.

## Figures and Tables

**Table 1:** List of antibodies used for EM immunolabelling. Antibodies name, host, target, dilution, and code are reported.

Antibody (Host)	Target	Dilution	Reference
Anti-5mC (mouse)	5mC	1:500 in PBS/BSA/TWEEN20	GTX629448–AB_2888114 (GeneTex, Irvine, CA, USA)
Anti-5hmC (mouse)	5hmC	1:150 in PBS/BSA/TWEEN20	GTX629765–AB_2736902 (GeneTex)
Anti-DNMT3a (rabbit)	DNMT3a	1:10 in PBS/BSA/TWEEN20	GTX129125–AB_2885902 (GeneTex)
Anti-TET2 (mouse)	TET2	1:20 in PBS/BSA/TWEEN20	GTX629881–AB_2888171 (GeneTex)
Anti-H3K9me3 (rabbit)	H3K9me3	1:20 in PBS/BSA/TWEEN20	GTX121677–AB_10721938 (GeneTex)
Anti-H3K9Ac (rabbit)	H3K9Ac	1:20 in PBS/BSA/TWEEN20	GTX88007–AB_10731164 (GeneTex)
Anti-H3K27me3 (rabbit)	H3K27me3	1:20 in PBS/BSA/TWEEN20	GTX121184–AB_10618572 (GeneTex)
Anti-Rabbit 12 nm gold particle conjugated (goat)	Rabbit IgG	1:20 in PBS	111-205-144– AB_2338016 (Jackson ImmunoResearch, West Grove, PA, USA)
Anti-Mouse 12 nm gold particle conjugated (goat)	Mouse IgG	1:20 in PBS	115-205-068– AB_2338730 (Jackson ImmunoResearch)

**Table 2:** List of antibodies used for STED immunolabelling. Antibodies name, host, target, dilution, and code are reported.

Antibody (Host)	Target	Dilution	Reference
Anti-EZH2 (rabbit)	EZH2	1:200 in PBS/BSA 1%	GTX110384–AB_2885283 (GeneTex)
Anti-NO-Tyr	Nitrotyrosine	1:200 in PBS/BSA 1%	sc-32757–AB_628022 (Santa Cruz Biotechnology, Dallas, TX, USA)
Alexa 568 (goat)	Rabbit IgG	1:100 in PBS/BSA 1%	A11036–AB_10563566 (Invitrogen, Waltham, MA, USA)
DyLight594 (goat)	Mouse IgG	1:100 in PBS/BSA 1%	GTX213111-05–AB_2887581 (GeneTex)

## Data Availability

Not applicable.

## Supplementary Materials

The following supporting information can be downloaded at: https://www.mdpi.com/article/10.3390/ijms24010153/s1.
