# Supplementary material for: Oxidative Stress and Nuclear Reprogramming: A Pilot Study of the Effects of Reactive Oxygen Species on Architectural and Epigenetic Landscapes
Source: Int J Mol Sci. 2022 Dec 21;24(1):153. doi: 10.3390/ijms24010153 (PMC9820425; doi:10.3390/ijms24010153)
Supplement: Supplementary file 1 [file ijms-24-00153-s001.zip › ijms-2054384-supplementary.pdf]

Oxidative Stress and Nuclear Reprogramming: a Pilot Study of the Effects of Reactive Oxygen Species on Architectural and Epigenetic Landscapes

Claudio Casali [1], Stella Siciliani [1], Luca Galgano [2] and Marco Biggiogera [1,*]

1 Laboratory of Cell Biology and Neurobiology, Department of Biology and Biotechnology "L. Spallanzani", University of Pavia, 27100 Pavia, Italy
2 Laboratory of Biochemistry, Department of Biology and Biotechnology "L. Spallanzani", University of Pavia, 27100 Pavia, Italy
* Correspondence: marco.biggiogera@unipv.it; Tel.: +39-0382-986327

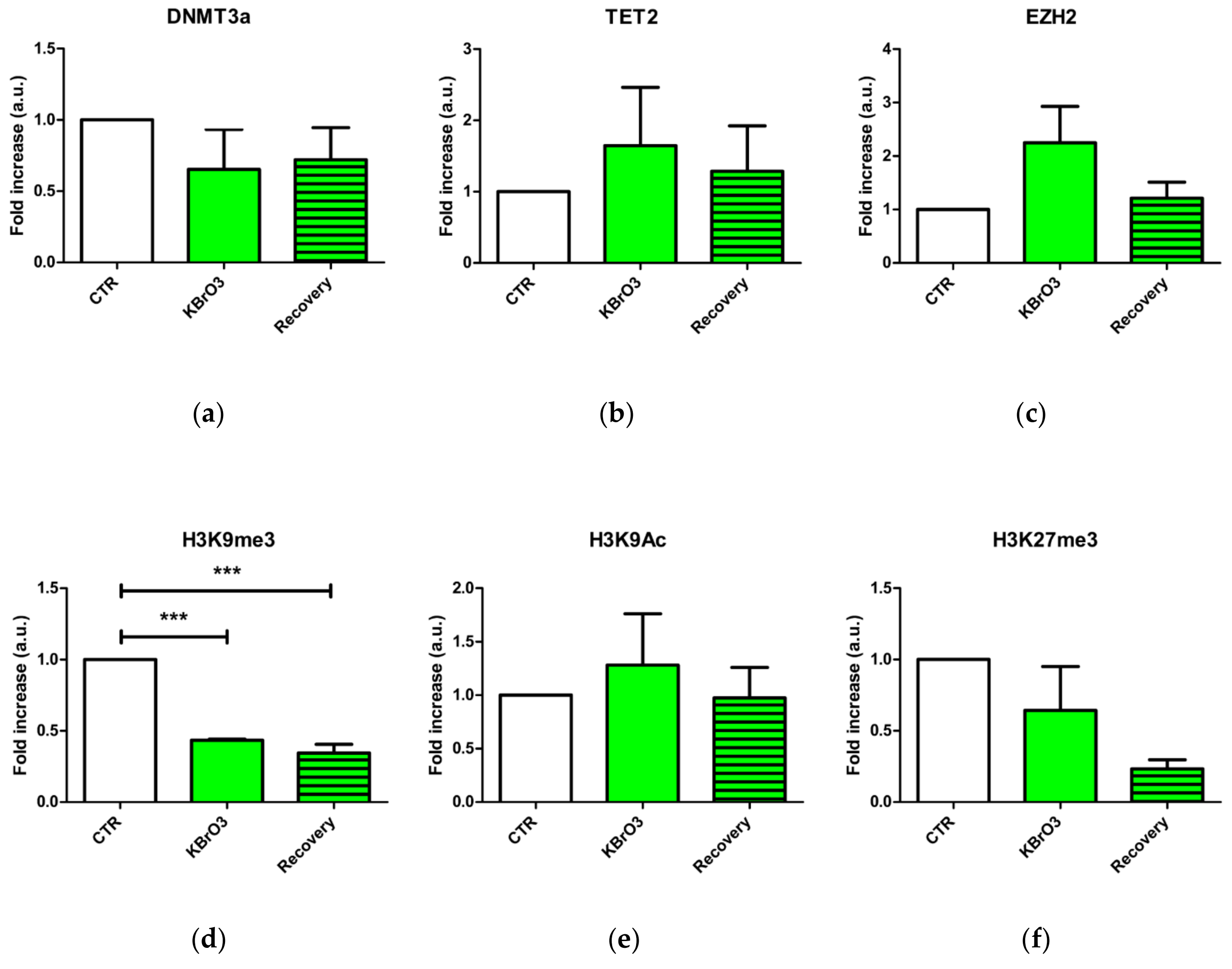

Figure S1. Western blot quantification and statistical analysis. (**a**) DNMT3a. (**b**) TET2. (**c**) EZH2. (**d**) H3K9Ac. (**e**) H3K9me3. (**f**) H3K27me3. Although significance is not always reached, mostly due to isolation of cytoplasmic fractions, these results confirm the same trend already described by means of electron microscopy (***: P value<0,001).

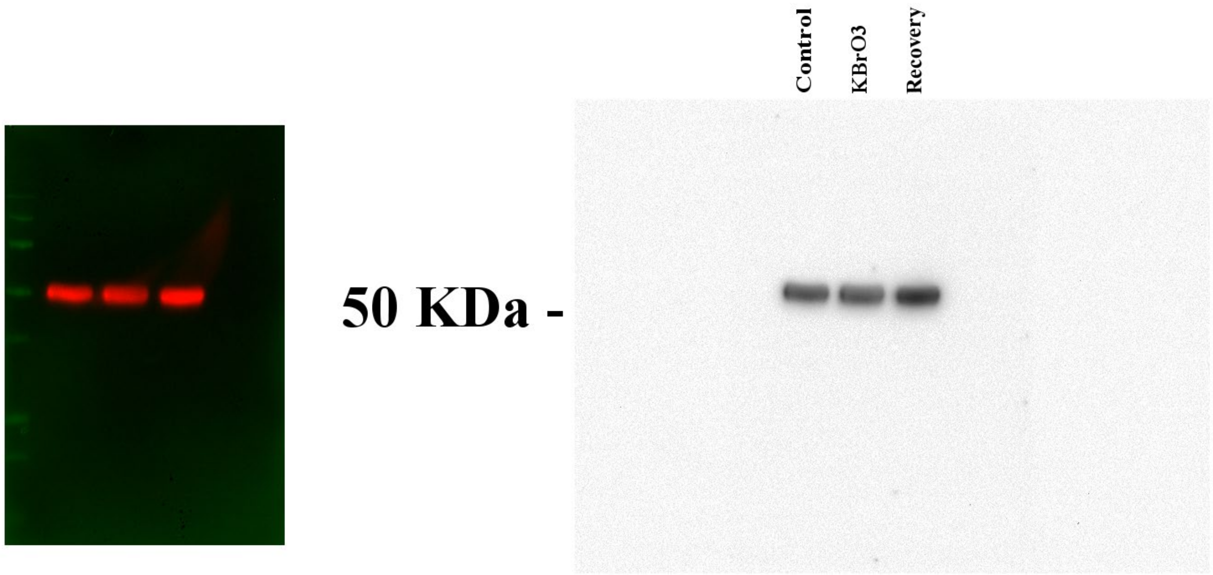

Figure S2. Western blot loading control. Tubulin was assessed as loading control with a molecular weight of 50 KDa as reference.
